# Revision of the genus *Reichardtiolus* Kryzhanovskij, 1959 (Coleoptera, Histeridae, Saprininae)

**DOI:** 10.3897/zookeys.379.6457

**Published:** 2014-02-11

**Authors:** Tomáš Lackner

**Affiliations:** 1 Czech University of Life Sciences, Faculty of Forestry and Wood Sciences, Department of Forest Protection and Entomology, Kamýcká 1176, CZ-165 21 Praha 6 – Suchdol, Czech Republic

**Keywords:** Coleoptera, Histeridae, Saprininae, *Reichardtiolus*, Palaearctic Region, taxonomic revision

## Abstract

The genus *Reichardtiolus* Kryzhanovskij, 1959 is revised herein. It now contains five species: *R. duriculus* (Reitter, 1904) from middle Asia (with a doubtful female specimen from western China that is here tentatively assigned to this species), *R. pavlovskii* Kryzhanovskij, 1959 from Turkmenistan, *R. sphingis* (Peyerimhoff, 1936), **comb. n.** (transferred from *Saprinus* Erichson, 1834) from Egypt and Jordan, *R. perses* **sp. n.** from Iran and *R. aldhaferi* **sp. n.** from Saudi Arabia. Except for *R. pavlovskii*, which is a rather distinct species known only from two females, the remaining species are allopatric, very similar externally and are best separated from each other by their male terminalia. *R. pavlovskii* is kept in *Reichardtiolus* only tentatively, pending the examination of more specimens, and especially its male genitalia. *R. duriculus* and *R. pavlovskii* are re-described, while *R. perses* **sp. n.**, *R. aldhaferi* **sp. n.** and *R. sphingis* **comb. n.** are provided with diagnostic descriptions because of their overall similarity with *R. duriculus*. Morphological differences of all species are illustrated using SEM micrographs. Male genitalia of *R. duriculus*, *R. sphingis* **comb. n.**, *R. perses* **sp. n.** and *R. aldhaferi* **sp. n.** are illustrated and a key to the species is given. *R. duriculus* is newly recorded from Tajikistan.

## Introduction

The genus *Reichardtiolus* was established by Kryzhanovskij (1959) based on the species *Saprinus duriculus* Reitter, 1904. At the time of its designation *Reichardtiolus* was a mere subgenus of the genus *Exaesiopus* Reichardt, 1926 and Kryzhanovskij (1959) included in it another species, *Reichardtiolus pavlovskii*, which he described in the same work. In their fauna of the USSR, Kryzhanovskij and Reichardt (1976) elevated the rank of *Reichardtiolus* from a subgenus of *Exaesiopus* to fully-fledged genus. Lackner (2010) summarized the knowledge about the genus without having examined the obscure and very rare taxon *Reichardtiolus pavlovskii*. During the years 2006–2013 I had the opportunity to examine a large number of Saprininae taxa, among them the rare *Reichardtiolus pavlovskii* and *Saprinus sphingis* Peyerimhoff, 1936, the latter of which has been treated as a species incertae sedis since its description (Peyerimhoff 1936; Mazur 1984; 1997; 2004; 2011). One undescribed species, apparently belonging to *Reichardtiolus* from Saudi Arabia was recently discovered in the collections of the King Saud Museum of Arthropods (KSMA), and the author’s visit to the Zoological Institute of the Russian Academy of Sciences (ZIN) yielded another new species from south-western Iran. The results of these examinations are presented below. This work presents another contribution to the on-going revisionary work of the genera of the subfamily Saprininae (Lackner 2009a-c, 2010, 2011a, b; Tishechkin and Lackner 2012; Lackner 2012; Lackner 2013a, b; Lackner and Gomy 2013).

## Material and methods

All dry-mounted specimens were relaxed in warm water for several hours or overnight, depending on the body size. After removal from original cards, the beetles were side-mounted on triangular points and observed under a Nikon 102 stereoscopic microscope with diffused light. Body structures were studied using methods described by Ôhara (1994): male genitalia were macerated in a hot 10% KOH solution for about 15 minutes, cleared in 80% alcohol, macerated in lactic acid with fuchsine, incubated at 60 °C for two hours, and subsequently transferred into a 1:1 mixture of glacial acetic acid and methyl salicylate, heated at 60 °C for 15 minutes and cleared in xylene. Specimens were then observed in α-terpineol in a small glass dish. Digital photographs of the male terminalia were taken by a Nikon 4500 Coolpix camera and edited in Adobe Photoshop CS4. Based on the photographs or direct observations, the genitalia were drawn using a light-box Hakuba klv-7000. SEM photographs of *Reichardtiolus duriculus*, *Reichardtiolus pavlovskii* and *Reichardtiolus sphingis* were taken with a JSM 6301F microscope at the laboratory of Faculty of Agriculture, Hokkaido University, Sapporo, Japan while those of *Reichardtiolus aldhaferi* and *Reichardtiolus perses* were taken at the Laboratory of the Electron Microscopy at the Faculty of Biology, Charles University, Prague, Czech Republic. All available specimens were measured with an ocular micrometer. Beetle terminology follows that of Ôhara (1994) and Lackner (2010). Separate lines of the same label are demarcated by a slash (/). The following acronyms of museums and private collections are used throughout the text:

CAS Alexander Sokolov collection, Moscow, Russia

CAT Alexey K. Tishechkin collection, Baton Rouge, Louisiana, USA

CND Nicolas Dégallier collection, Paris, France

CPV Pierpaolo Vienna collection, Venice, Italy

CYG Yves Gomy collection, Nevers, France

FMNH Field Museum of Natural History, Chicago, USA (J. Boone)

HNHM Hungarian Natural History Museum, Budapest, Hungary (O. Merkl)

KSMA King Saud Museum of Arthropods, Riyadh, Saudi Arabia (H. M. Al Dhafer)

MSNG Museo Civico di Storia Naturale “Giacomo Doria”, Genoa, Italy (M. Tavano)

TLAN Tomáš Lackner collection, temporarily housed at Naturalis Biodiversity Centre, Leiden, Netherlands

ZIN Zoological Institute, Russian Academy of Sciences, St. Petersburg, Russia (B. Kataev)

**Abbreviations.** Abbreviations of morphological measurements follow Ôhara (1994) and are used throughout the text as follows:

APW width between anterior angles of pronotum

EL length of elytron along elytral suture

EW maximum width between outer margins of elytra

PEL length between anterior angles of pronotum and apices of elytra

PPW width between posterior angles of pronotum

## Taxonomy

### Reichardtiolus

Kryzhanovskij, 1959

http://species-id.net/wiki/Reichardtiolus

Reichardtiolus Kryzhanovskij, 1959: 217 (as a subgenus of *Exaesiopus*). Type species *Saprinus duriculus* Reitter, 1904, original designation.
Reichardtiolus : Kryzhanovskij and Reichardt (1976): 112, 238; Mazur (1984): 103; Mazur (1997): 265; Mazur (2004): 96; Lackner (2010): 63, 186; Mazur (2011): 210.

#### Diagnosis.

**Figure 1.**
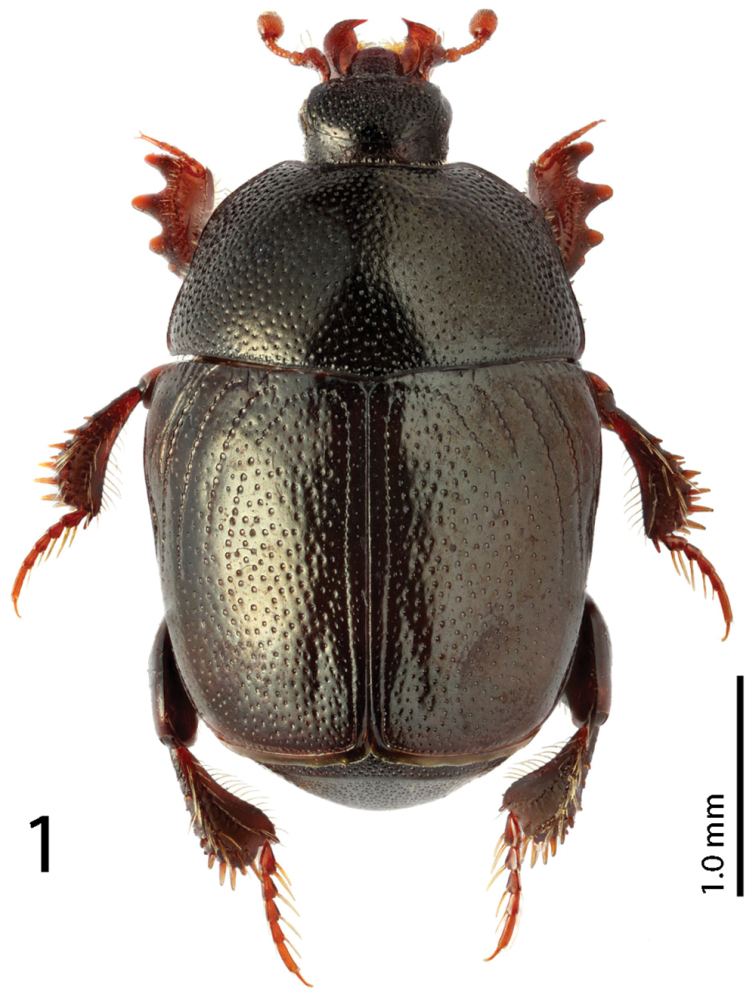
*Reichardtiolus duriculus* (Reitter, 1904) habitus. (Photo by M. Smirnov, Ivanovo, Russia).

**Figures 2–9.**
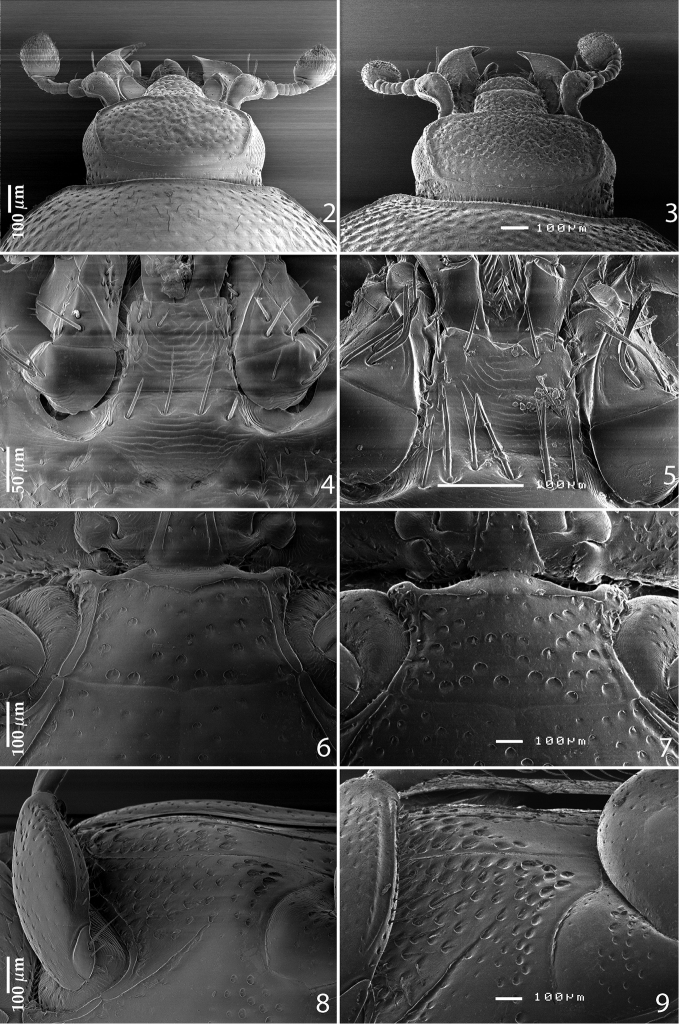
**2** *Reichardtiolus duriculus* (Reitter, 1904) head, dorsal view **3** *Reichardtiolus sphingis* (Peyerimhoff, 1936), comb. n., head, dorsal view **4** *Reichardtiolus duriculus* (Reitter, 1904) mentum, ventral view **5** *Reichardtiolus sphingis* (Peyerimhoff, 1936), comb. n., mentum, ventral view **6** *Reichardtiolus duriculus* (Reitter, 1904) mesoventrite **7** *Reichardtiolus sphingis* (Peyerimhoff, 1936), comb. n., mesoventrite **8** *Reichardtiolus duriculus* (Reitter, 1904) lateral disk of metaventrite **9** *Reichardtiolus sphingis* (Peyerimhoff, 1936), comb. n., lateral disk of metaventrite.

**Figures 10–13.**
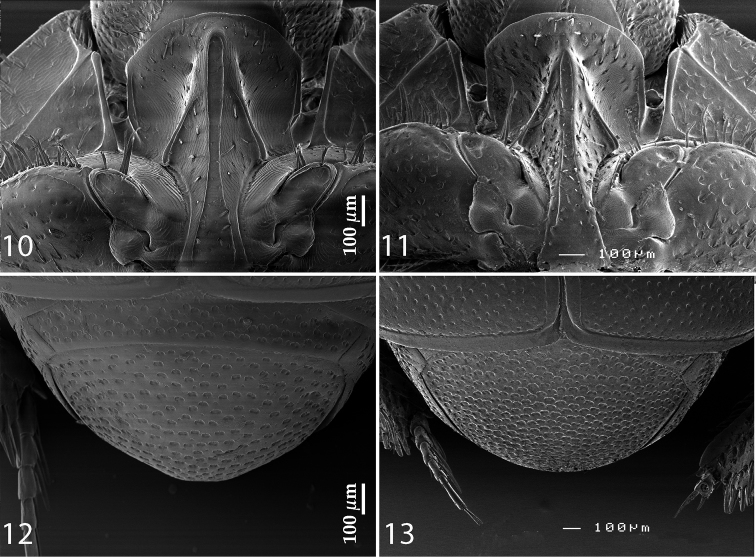
**10** *Reichardtiolus duriculus* (Reitter, 1904) prosternum **11** *Reichardtiolus sphingis* (Peyerimhoff, 1936), comb. n., prosternum **12** *Reichardtiolus duriculus* (Reitter, 1904) pygidium **13** *Reichardtiolus sphingis* (Peyerimhoff, 1936), comb. n., pygidium.

*Reichardtiolus* has been recently diagnosed by Lackner (2010), but the published diagnosis has to be adapted with respect to the newly examined *Reichardtiolus pavlovskii*, *Reichardtiolus sphingis*, *Reichardtiolus perses* and *Reichardtiolus aldhaferi* as follows: body size 2.00–4.25 mm, cuticle (Fig. 1) chestnut brown to almost black with or without slight metallic tinge or lustre; frontal stria (Figs 2, 3) usually weakened medially, but may be complete to widely interrupted (in *Reichardtiolus pavlovskii*); frons variously densely punctuate, punctures separated by less than half their diameter to twice their diameter; occasionally with protuberances or shallow depressions; clypeus rectangular to rounded, occasionally margined, anterior margin may be elevated; dorsal surface densely to very densely and coarsely punctuate, punctures separated by their own to half their own diameter, in *Reichardtiolus pavlovskii* even forming longitudinal wrinkles on pronotum (Fig. 62); pronotal depressions absent; dorsal elytral striae in *Reichardtiolus pavlovskii* almost unrecognizable beneath coarse punctuation, in other congeners usually all four dorsal elytral striae 1–4 well discernible; prosternal foveae present (Fig. 10) or absent (*Reichardtiolus pavlovskii*; Fig. 68); prosternal process often compressed, concave or convex, especially on posterior half, punctate and setose; both sets of prosternal striae present (in case of *Reichardtiolus pavlovskii* only as vague rudiments); pronotal hypomeron, lateral disc of metaventrite and metepisternum setose. Protibia (Figs 1, 64) with two or three short teeth each topped by variably large denticle, usually followed by one or two much smaller denticles entombed in outer margin of protibia; meso- and metafemora strongly thickened (Fig. 63); metatibia dilated and thickened; anterior surface of metatibia with two to several rows of short, stout denticles (Fig. 71).

#### Differential diagnosis.

Members of *Reichardtiolus* are externally most similar to the species of the genus *Exaesiopus* Reichardt, 1926, differing from them especially by the absence of deep longitudinal rugae on the frontal disc. The elytra in *Reichardtiolus* are entirely coarsely and densely punctate, in *Reichardtiolus pavlovskii* even forming rugulose-lacunose wrinkles, whereas in *Exaesiopus* the elytra are always at least partly glabrous. Because of the thickened hind femora and lack of longitudinal furrows on frons, *Reichardtiolus* cannot be confused with any other Palaearctic taxon; for further details on differential diagnosis and a key to genera of the Palaearctic Histeridae the reader is referred to Lackner (2010).

#### Biology.

*Reichardtiolus* is a psammophilous taxon, found in arid and desert habitats, often in sand or under decaying vegetation (Lackner 2010); several specimens of *Reichardtiolus aldhaferi* and *Reichardtiolus duriculus* were also collected at light or in rodent’s burrows. According to Kryzhanovskij in Kryzhanovskij and Reichardt (1976) the second known specimen of *Reichardtiolus pavlovskii* was collected while digging in sands under *Tamarix*.

#### Distribution.

*Reichardtiolus duriculus* is found across middle Asia: Kazakhstan, Turkmenistan, Uzbekistan and Tajikistan, with a female specimen recorded from western China that I here tentatively assign to this species (Lackner 2010; Mazur 2011); *Reichardtiolus pavlovskii* is known currently only from eastern Turkmenistan, *Reichardtiolus sphingis* has been collected in southern Jordan and northern Egypt. Two newly described species, *Reichardtiolus aldhaferi* sp. n. and *Reichardtiolus perses* sp. n., are known only from the environs of Riyadh, Saudi Arabia and environs of Kerman, south-western Iran, respectively (Fig. 72).

### Reichardtiolus duriculus

(Reitter, 1904)

http://species-id.net/wiki/Reichardtiolus_duriculus

Figs 1 , 2 , 4 , 6 , 8 , 10 , 12 , 14–23

Saprinus duriculus Reitter, 1904: 31.
Styphrus duriculus : Jakobson (1911): 651.
Hypocacculus duriculus : Bickhardt (1916): 97.
Exaesiopus duriculus : Reichardt (1926): 17; Reichardt (1941): 330, 333, Fig. 172.
Reichardtiolus duriculus : Kryzhanovskij and Reichardt (1976): 239, Figs 465, 466, 468; Mazur (1984): 103; Mazur (1997): 265; Mazur (2004): 96; Lackner (2010): 187, Figs 27, 67, 132, 593–610; Mazur (2011): 210.

#### Type locality.

Turkmenistan, Mary.

#### Type material examined.

Holotype: ♀, side-mounted on a triangular point, four segments of meso-tarsomere broken off, last two meta-tarsomeres broken off, with the following labels: “♀” [printed]; followed by: “Merw” [printed]; followed by: “Ahnger” [printed]; followed by: “*Styphrus duriculus* / m. 1904 Typ” [written label]; followed by: “coll. Reitter” [printed]; followed by: “1960 / *Exaesiopus* / (*Reichardtiolus*) / *duriculus* Rchdt (sic!) / Kryzhanovskij det.” [printed-written]; followed by: “Holotypus 1904 / *Saprinus* / *duriculus* / Reitter” [red-framed printed-written label] (HNHM).

#### Additional material examined.

**Turkmenistan:** 1 ♂, Anau, Karakum, 21.iv.1981, A. Olexa lgt.; 1 ♀ & 1 spec., Repetek, 12.iv.1989, M. Nikodým lgt.; 1 ♀, Amurdarja-Kirki, 1.-5.v.1993, no collector (all exs. TLAN); 1 spec., Karakum, Repetek, 4.v.1983, Krivoshatsky lgt., at light; 1 spec., Tschardshou, Repetek, 14.iv.1983, Snížek lgt. (both CPV); 4 specs., ibid, but MSNG; 1 spec., Repetek, in burrow of *Rhombomys opimus*, 1.iv.1980, Krivoshatskij lgt. (ZIN); 1 spec., ibid, but 19.iv.1982, at light, same collector (ZIN); 1 spec., 20 km E of Kerka, 23.iv.1984, at light, T. Vereschagina lgt. (ZIN). **KAZAKHSTAN:** 1 ♀, Temir env., river Chatryly, 26.v.1908, D. Borodin & B. Uvarov lgt. (ZIN); 2 specs., Mangyshlak peninsula, Schtepe env., 24.-27.iv.1999, Smirnov leg (CAS); 1 spec., without further data (MSNG); 1 spec., low Ili River, env. Bakanas, 15.iv.1971, Badenko lgt. (ZIN); 1 spec., Gurivskaya oblast, Makata distr., prom. Iskair, 13.vi.1981, Saraev lgt. (ZIN). **UZBEKISTAN:** 1 ♀, Syr-Darya gebiet, Perovsk uezd, 5.v.1905, J. Baeckmann lgt. (ZIN); 1 ♀, Kyzyl-Kum, Yny-Darja, Perovsk uezd, 24.iv.1911, Ivanov lgt. (ZIN); 2 specs., Kyzyl-Kum, Ayak-Agytma, 20.iv.1965, G. Medvedev lgt., sands (ZIN); 1 spec., Kyzyl-Kum, 70 km S of Tamdy, 1.v.1965, L. Arnoldi lgt. (ZIN). **Tajikistan:** 1 ♀, Syr-Daria Riv., nr. Karakum Reservoir, at 40°32'16''N, 70°17'47''E, 13.iv.61, sandy desert, I.K.Lopatin lgt. (CAT). **CHINA:** 1 ♀, Xinjiang Prov., mountain range Tokuz-Daban, upper Cherchen [=Qarqan] River, v. [18]90, Pevtzov lgt. (with doubt) (ZIN).

#### Re-description.

Although this species has been recently re-described by the author (Lackner 2010: 187), and the reader is referred there for the exhaustive account of SEM micrographs and drawings of the mouthparts and sensory structures of the antenna, I prefer to repeat its re-description here for the reason that the following three species (*Reichardtiolus sphingis*, *Reichardtiolus aldhaferi* and *Reichardtiolus perses*) are morphologically very similar to *Reichardtiolus duriculus*. Those species are consequently provided only with diagnostic descriptions illuminating their respective differences from *Reichardtiolus duriculus*.

Body length: PEL: 2.00–3.40 mm; APW: 0.65–1.05 mm; PPW: 1.375–2.40 mm; EL: 1.25–2.25 mm; EW: 1.50–2.70 mm. Body (Fig. 1) elongate oval, strongly convex, cuticle dark brown with feeble metallic luster; legs, antennae and mouthparts rufous. Antennal scape (for fig. see Lackner 2010, fig. 596) slightly thickened, with several short setae; club (for fig. see Lackner 2010, fig. 595) rather large, without visible articulation, apical four-fifths covered with short sensilla intermingled with longer sparse erect sensilla, basal fifth glabrous; sensory structures of antennal club (for fig. see Lackner 2010, fig. 27) in form of stipe-shaped vesicle situated under circular sensory area on internal distal margin of the ventral side of antennal club.

Mouthparts: mandibles (for fig. see Lackner 2010, fig. 101) with rounded outer margin, strongly curved inwardly, mandibular apex acutely pointed; sub-apical tooth on inner margin of left mandible blunt; labrum (for fig. see Lackner 2010, fig. 67) convex, coarsely punctate; with two labral pits, each with two well-sclerotized setae; terminal labial palpomere thickened, its width about half its length; mentum (Fig. 4) sub-trapezoidal, anterior margin shallowly emarginate medially; antero-lateral corners with few short setae, lateral margins with a single row of short ramose setae; disc of mentum imbricate, asetose; cardo of maxilla with few short setae on lateral margin; stipes triangular, with three short setae; terminal maxillary palpomere thickened, its width about half its length, about twice as long as penultimate.

Clypeus (Fig. 2) slightly concave medially, rounded laterally, rugulose-lacunose; frontal stria well impressed, carinate, almost straight, somewhat weakened medially, continued as well-impressed, carinate supraorbital stria; frontal disc (Fig. 2) densely punctate; eyes slightly convex, visible from above.

Pronotum (Fig. 1) convex, pronotal sides rounded, convergent anteriorly on their apical third, apical angles inconspicuous; marginal pronotal stria complete, carinate; disc with very deep, dense and coarse punctures, laterally rugulose-lacunose, medially punctuation weakens and becomes sparser; pronotal hypomeron with sparse short amber setae.

Elytral epipleuron with a row of deep punctures; marginal epipleural stria well impressed, complete; marginal elytral stria complete, deeply impressed, carinate, continued as complete apical elytra stria. Humeral elytral stria weakly impressed on basal third, often doubled; inner subhumeral stria inconspicuous, present as tiny median fragment; elytra with four dorsal striae 1–4, in large punctures, first, second and third dorsal striae about the same length, reaching approximately elytral half apically, fourth dorsal elytral stria weakly impressed on basal third (occasionally longer apically), connected to complete sutural elytral stria. Elytral disc with deep round punctuation, punctures separated by 2–4 times their diameter, becoming finer apically and laterally; between sutural elytral stria and elytral suture a row of regular fine punctures present.

Propygidium transverse, coarsely and densely punctate; pygidium (Fig. 12) almost as long as broad, with sparser punctuation; interspaces in both cases finely imbricate.

Anterior margin of median portion of prosternum (Fig. 10) rounded; marginal prosternal stria present laterally and as vague anterior fragment; prosternal foveae rather small; prosternal process rather narrow, slightly concave; carinal prosternal striae slightly carinate, almost parallel, united in front of strongly carinate, shortened lateral prosternal striae. Surface between carinal prosternal striae almost smooth, prosternal apophysis with several microscopic setae; lateral parts of prosternal process strigulate with scattered microscopic punctures fringed with tiny setae.

Anterior margin of mesoventrite (Fig. 6) feebly emarginate medially; discal marginal mesoventral stria well-impressed, carinate, slightly weakened anteriorly; disc of mesoventrite with scattered deep, round punctures, fringed with microscopic setae; meso-metaventral sutural stria absent; meso-metaventral suture distinct.

Intercoxal disc of metaventrite slightly longitudinally concave in male, with coarse scattered punctures, area around lateral metaventral stria smooth; lateral metaventral stria (Fig. 8) deeply impressed, carinate, extending obliquely and shortened apically; lateral disc of metaventrite (Fig. 8) with shallow large setiferous punctures; metepisternum on basal half with similar punctuation, apical half of metepisternum (Fig. 8) almost smooth, fused metepimeron with few punctures; metepisternal stria present along entire fused metepimeron and metepisternum, intermittent basally.

Intercoxal disc of first abdominal sternite completely striate laterally, with sparse coarse punctuation.

Protibia (for fig. see Lackner 2010, fig. 603) flattened and somewhat dilated, apical protibial margin formed by anterior margin of large sub-triangular distal-most tooth topped with large triangular denticle, outer margin apart from this tooth with another similar tooth topped with large triangular denticle, followed by another, much lower tooth topped by much smaller triangular denticle and another microscopic denticle entombed in outer margin of protibia; setae of outer row on anterior surface of protibia sparse, regular and short; setae of intermediate row similarly sparse and regular, much shorter than those of outer row; protarsal groove moderately deep; anterior protibial stria present only on basal third; tarsal denticles absent; protibial spur tiny, bent, growing out from apical protibial margin; apical margin of protibia posteriorly without denticles; outer part of posterior surface of protibia sparsely punctate, distinctly separated from glabrous median part of posterior surface by irregular costiform stria fringed with sparse microscopic setae; posterior protibial stria complete, deeply impressed, with sparse microscopic setae; inner-ventral denticles absent; inner margin with single row of well sclerotized setae.

Mesotibia (for fig. see Lackner 2010, fig. 601) slightly thickened, outer margin with two sparse rows of thin denticles greater in size apically; setae of outer row rather dense, strongly sclerotized and longer than denticles of outer margin; setae of intermediate row sparse, microscopic; posterior mesotibial stria inconspicuous; anterior surface of mesotibia imbricate, with scattered minuscule punctures with microscopic setae; anterior mesotibial stria shortened apically, almost complete; mesotibial spur stout, rather short; apical margin with several tiny denticles; claws of apical tarsomere longer than half its length; metatibia basically similar to mesotibia, but much more thickened and dilated, rows of denticles of outer margin widely separated, outer row of denticles (for fig. see Lackner 2010, fig. 602) observable only from ventral view.

**Figures 14–23.**
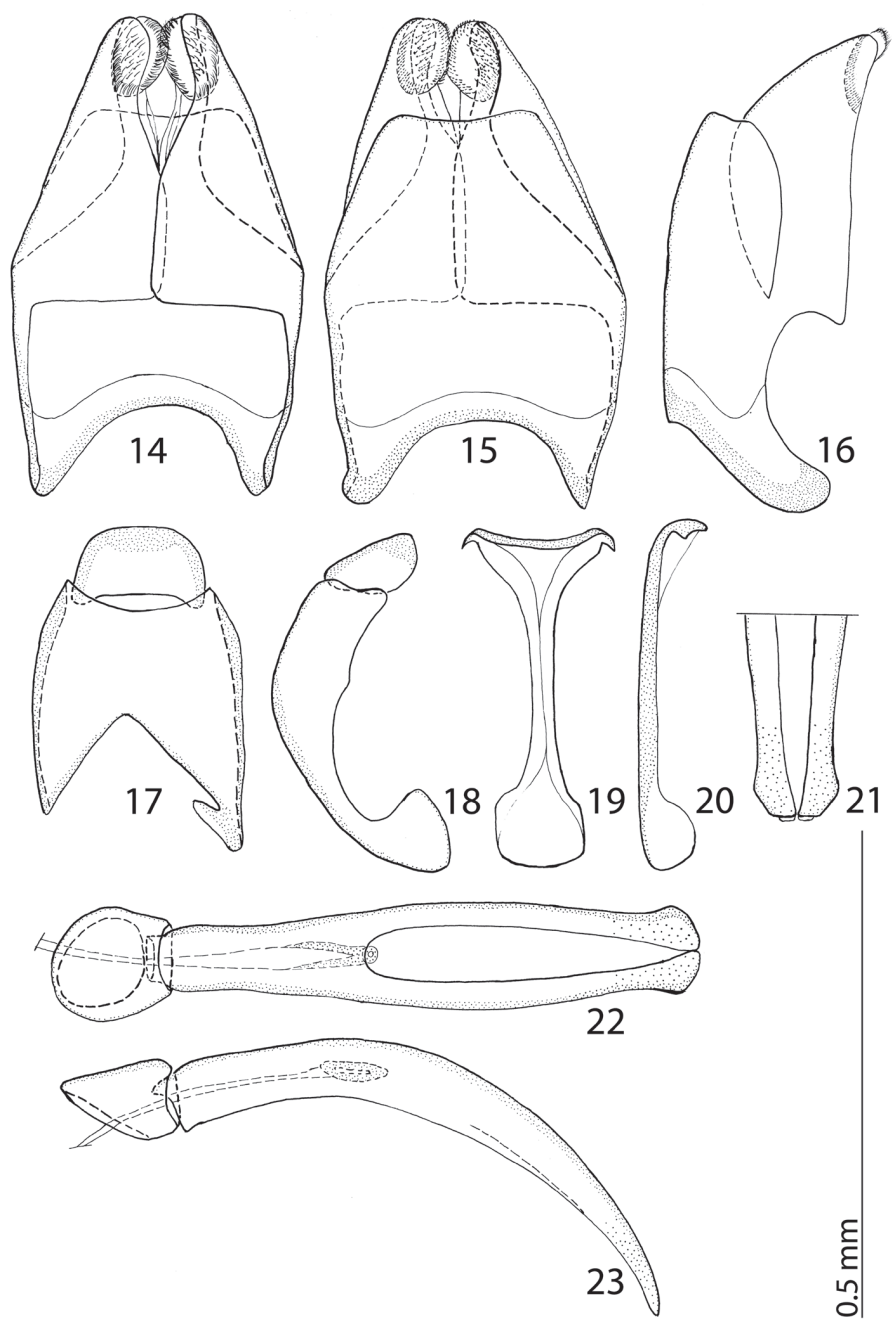
**14** *Reichardtiolus duriculus* (Reitter, 1904) 8^th^ sternite and tergite, ventral view **15** ditto, dorsal view **16** ditto, lateral view **17** *Reichardtiolus duriculus* (Reitter, 1904) 9^th^ + 10^th^ tergites, dorsal view **18** ditto, lateral view **19** *Reichardtiolus duriculus* (Reitter, 1904) spiculum gastrale, ventral view **20** ditto, lateral view **21** *Reichardtiolus duriculus* (Reitter, 1904) apex of aedeagus, frontal view **22** *Reichardtiolus duriculus* (Reitter, 1904) aedeagus, dorsal view **23** ditto, lateral view.

Male genitalia: Eighth sternite (Figs 14–15) divided medially, apically with short setae and a setose velum, 8^th^ tergite apically only faintly emarginate, 8^th^ sternite and tergite fused laterally, deep from lateral view (Fig. 16). Tenth tergite (Fig. 17) basally almost straight; 9^th^ tergite apically inwardly arcuate, anterior angles prominent (Fig. 17), sclerotization not divided medially. Spiculum gastrale (Figs 19–20): tips on anterior end without strong sclerotization, posterior end outwardly arcuate. Basal piece of aedeagus (Figs 22–23) rather short, ratio to tegmen 1:5; aedeagus tube-like, with large opening for median lobe, apically with numerous pseudopores, curved laterally (Fig. 22); apex of aedeagus blunt (Fig. 21).

#### Differential diagnosis.

*Reichardtiolus duriculus* is most readily separated from *Reichardtiolus pavlovskii* from which it differs by the body size and other substantial morphological characters, e.g. the presence (vs. absence) of prosternal foveae, presence of elytral striae (almost indiscernible in *Reichardtiolus pavlovskii*) etc. The differences among *Reichardtiolus duriculus* and other three congeners are subtler and the species are best separated by their male terminalia; the reader is referred to the key to species for details.

#### Biology.

A psammophilous species, usually collected in sand, occasionally collected also in rodent’s burrows or even at light.

#### Distribution.

Turkmenistan, Kazakhstan, Uzbekistan, western China (?). Newly recorded from Tajikistan (Fig. 72).

#### Remarks.

The single specimen from Xinjiang is a female, and differs from the specimens from ex-Soviet middle Asia especially by very coarsely and rugosely punctate frons and clypeus, as well as denser and coarser punctuation of mesoventrite and pygidium. However, I am hesitant to describe a new species based on a single female and prefer rather keeping it tentatively as a specimen of *Reichardtiolus duriculus*. Certainly, acquisition of new material containing male specimens from the above-mentioned locality would help clarify its taxonomic status.

### Reichardtiolus sphingis

Peyerimhoff, 1936 comb. n.

http://species-id.net/wiki/Reichardtiolus_sphingis

Figs 3 , 5 , 7 , 9 , 11 , 13 , 24–33

Saprinus sphingis Peyerimhoff, 1936: 221; Mazur 1984: 64; Mazur 1997: 232; Mazur 2004: 101; Mazur 2011: 188.

#### Type locality.

Egypt, Sakkara.

#### Material examined.

**Egypt:** 1 ♀, Gebel Asfar, 2.iv.1935, coll. Alfieri Egypt (FMNH). **Jordan:** 2 ♂♂, 1 ♀ & 9 specs., 60 km N El Mudawwara, 1000 m, 29°20'N, 35°32'E, 5.iv.1994, Bečvář J. & S. lgt. (TLAN); 1 ♂, ibid, but CAT; 1 ♂, ibid, but CND; 10 ♀♀, ibid, but MSNG, 1 ♂ & 1♀, ibid, but CYG.

#### Diagnostic description.

**Figures 24–33.**
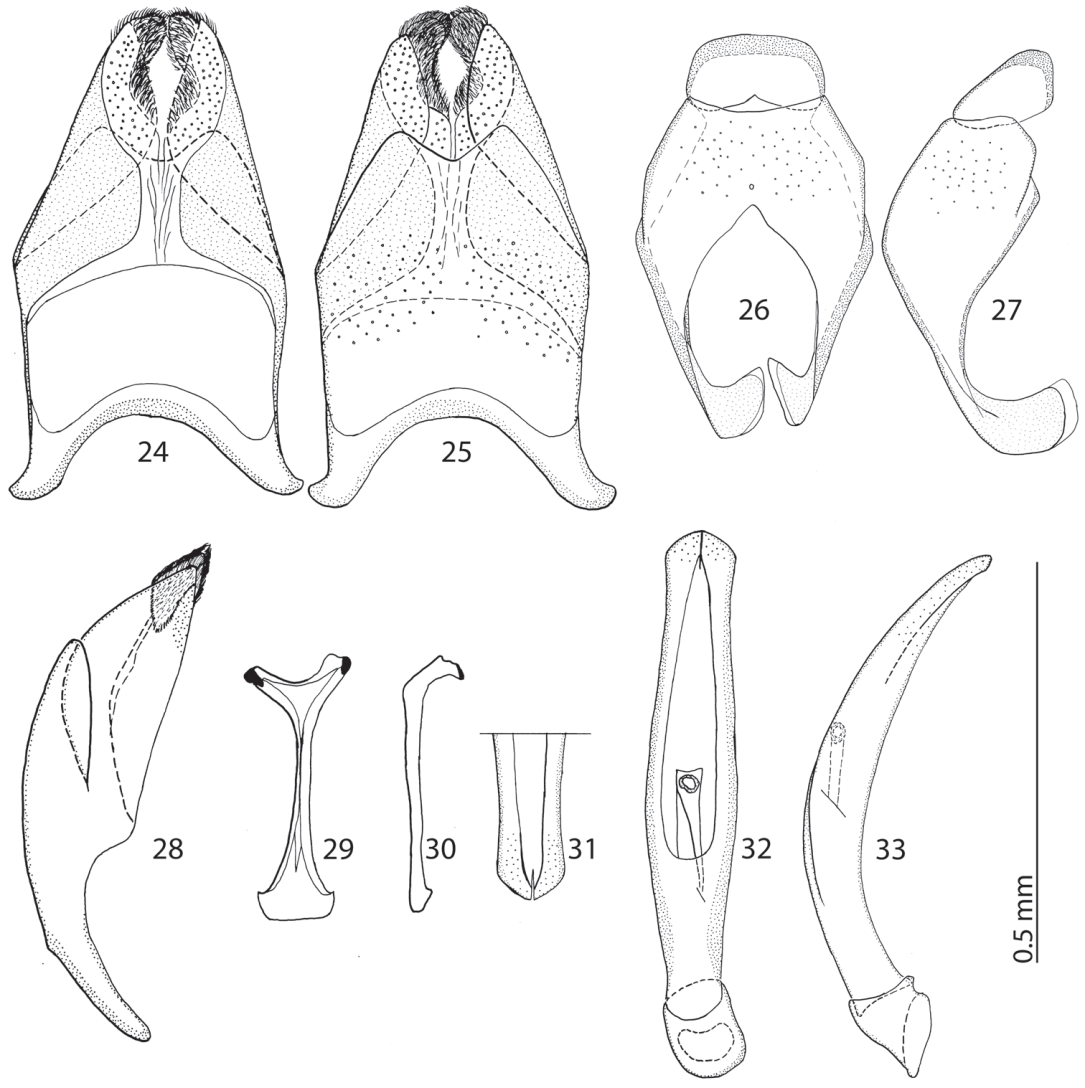
**24** *Reichardtiolus sphingis* (Peyerimhoff, 1936), comb. n., 8^th^ sternite and tergite, ventral view **25** ditto, dorsal view **26** *Reichardtiolus sphingis* (Peyerimhoff, 1936), comb. n., 9^th^ + 10^th^ tergites, dorsal view **27** ditto, lateral view **28** *Reichardtiolus sphingis* (Peyerimhoff, 1936), comb. n., 8^th^ sternite and tergite, lateral view **29** *Reichardtiolus sphingis* (Peyerimhoff, 1936), comb. n., spiculum gastrale, ventral view **30** ditto, lateral view **31** *Reichardtiolus sphingis* (Peyerimhoff, 1936), comb. n., apex of aedeagus, frontal view **32** *Reichardtiolus sphingis* (Peyerimhoff, 1936), comb. n., aedeagus, dorsal view 33 ditto, lateral view.

Body size: PEL: 2.80–3.25 mm; APW: 0.90–1.10 mm; PPW: 2.00–2.40 mm; EW: 2.25–2.65 mm; EL: 1.75–2.10 mm. Body as in *Reichardtiolus duriculus*, pronotum darker than elytra; legs, antennae and mouthparts rufous; antennae as in *Reichardtiolus duriculus*. Mouthparts as in *Reichardtiolus duriculus*, but mentum on its anterior margin with deeper emargination (compare Figs 4 and 5). Clypeus and frons similar to *Reichardtiolus duriculus* (compare Figs 2 and 3), but punctuation coarser and denser. Structure of pronotum and elytra similar to those of *Reichardtiolus duriculus*; punctuation of elytral disk somewhat sparser than that of *Reichardtiolus duriculus*. Propygydium and pygydium more coarsely punctate than those of *Reichardtiolus duriculus*, otherwise similar to it (compare Figs 12 and 13). Prosternum similar to that of *Reichardtiolus duriculus*, but more densely punctate (compare Figs 10 and 11). Mesoventrite similar to that of *Reichardtiolus duriculus*, but marginal mesoventral stria of *Reichardtiolus sphingis* anteriorly interrupted medially and rather straight (compare Figs 6 and 7). Metaventrite similar to that of *Reichardtiolus duriculus*, but lateral disk of metaventrite and metepisternum more coarsely punctate than those of *Reichardtiolus duriculus* (compare Figs 8 and 9). Abdominal ventrites similar to those of *Reichardtiolus duriculus*. Legs similar to those of *Reichardtiolus duriculus*, but teeth of protibia of *Reichardtiolus sphingis* more blunt than those of *Reichardtiolus duriculus* and denticles of meso- and metatibia of *Reichardtiolus sphingis* shorter, thinner and more blunt than those of *Reichardtiolus duriculus*. Male genitalia: 8^th^ sternite (Figs 24–25) well sclerotized, apically with small setose velum covered with pores; 8^th^ tergite (Fig. 25) apically widely emarginated medially, covered with pores and pseudopores. 9^th^ tergite (Fig. 26) strongly sclerotized laterally, anterior half with pores and pseudopores, laterally with projection (Fig. 27); basal margin of 10^th^ tergite inwardly arcuate (Fig. 26). Spiculum gastrale (Fig. 29) on anterior end strongly sclerotized on both tips; posterior end almost straight. Aedeagus of *Reichardtiolus sphingis* similar to that of *Reichardtiolus perses* (compare Figs 32–33 and 60–61); aedeagal apex of *Reichardtiolus perses* blunt, whereas pointed in *Reichardtiolus sphingis* (compare Figs 31 and 58).

#### Differential diagnosis.

*Reichardtiolus sphingis* is best separated from *Reichardtiolus pavlovskii* by the same characters as *Reichardtiolus duriculus*; for the differences among rest of the congeners the reader is referred to the key to species.

#### Biology.

According to Mr. S. Bečvář (pers. comm.) the series of this species from Jordan (El Mudawwara) was found under the grass at the foot of a small sand dune.

#### Distribution.

Egypt, surroundings of Cairo; south Jordan, 60 km N El Mudawwara (Fig. 72).

#### Remarks.

Peyerimhoff (1936) based his description of *Saprinus sphingis* on a single female, collected on 12 January 1933 in Sakkara, which is in northern Egypt (Peyerimhoff’s original description mentions “Basse-Egypte”), vicinity of Cairo. The type specimen was, according to Peyerimhoff’s description deposited in Alfieri’s collection. Although this collection has been (partly?) acquired by FMNH, the only specimen of *Saprinus sphingis* found there did not bear the locality labels corresponding with those of the Peyerimhoff’s type specimen. Therefore this specimen cannot be designated as the Lectotype and the type specimen of *Saprinus sphingis* remains undiscovered. However, the specimen treated here was most likely identified by Peyerimhoff as *Saprinus sphingis* and completely agrees with Peyerimhoff’s description. It has been collected near Jebel Asfar, which is north of Cairo. This locality is not far from Sakkara, which is south of Cairo. The specimens collected in southern Jordan by Mrs. J. & S. Bečvář (České Budějovice, Czech Republic) are virtually identical to the specimen from Egypt. Because the only known specimen of *Reichardtiolus sphingis* from Egypt is a female, the genitalia depicted in this work belong to one of the Jordanian specimens.

### Reichardtiolus aldhaferi sp. n.

http://zoobank.org/5DBC0C28-18FC-40FA-92B4-21222C33DE98

http://species-id.net/wiki/Reichardtiolus_aldhaferi

Figs 34 –47

#### Type locality.

Saudi Arabia, environs of Riyadh, Rhodet Khorim.

#### Type material examined.

Holotype, male, side-mounted on a triangular point with male genitalia extracted, dismembered and glued to the same mounting-point as the specimen, with following labels: “♂” (printed); followed by: “Saudi Arabia, Rhodet Khorim / 25°25.943'N, 47°13.863', Alt. / 572m 5.ii.2012 HP (B)” (printed, black-margined label); followed by: “*Reichardtiolus aldhaferi* / sp. n. Det. T. Lackner / 2013 HOLOTYPE” (red label, printed) (KSMA). Paratypes: 3 ♂♂ & 1 ♀, idem as Holotype (1 ♂ and 1 ♀ are sputter-coated with gold); 2 ♀♀, with following labels: “♀” (printed), followed by: “Saudi Arabia, Rhodet Khorim / N: 25°22'58" / E:47°16'44" / 08.i.2012 Light Trap (A) (printed, black-margined label); 1 ♀, with following labels: “♀” (printed), followed by: “Saudi Arabia Rhodet Khorim / N: 25°25'94"/ E: 47°13'86" / 25.xii.2011 Light Trap (B) (printed, black-margined label); 1 ♀, with following labels: “♀” (printed), followed by: “Saudi Arabia Kharah, Al / Mozahmiah 30km W.Riyadh / 24.ii.2011/LT / 28°23'33"N, 46°14'39"E / Al Dhafer, H.; Kondratieff,B.; / Fadl, H.&Al Gharbawi, A. (printed-written, black-margined label); 1 ♀, with following labels: “♀” (printed), followed by: KSA: Riyadh: Dirab / 20.i.1986 LT (written). All exs. KSMA except for 1 ♂ from Rhodet Khorim, 5.ii.2012 and 1 ♀, ibid, but 25.xii.2011 in coll. TLAN.

#### Diagnostic description.

**Figures 34–37.**
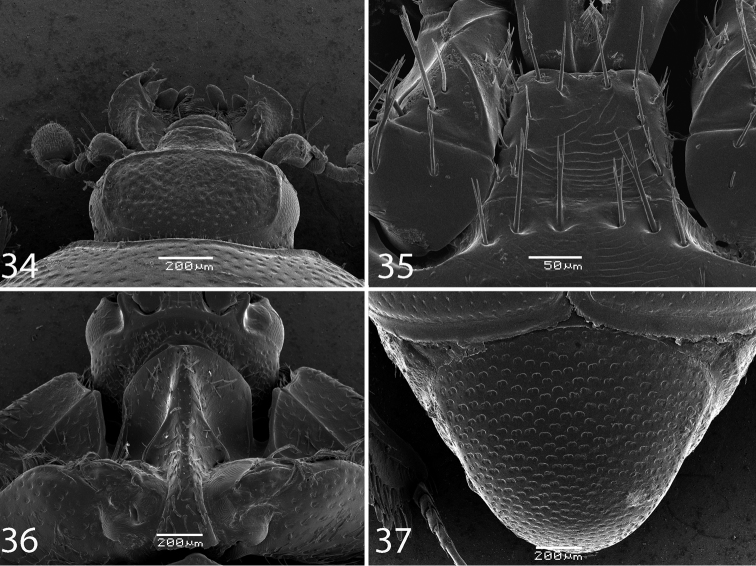
**34** *Reichardtiolus aldhaferi* sp. n., head, dorsal view **35** *Reichardtiolus aldhaferi* sp. n., mentum, ventral view **36** *Reichardtiolus aldhaferi* sp. n., prosternum **37** *Reichardtiolus aldhaferi* sp. n., pygidium.

**Figures 38–47.**
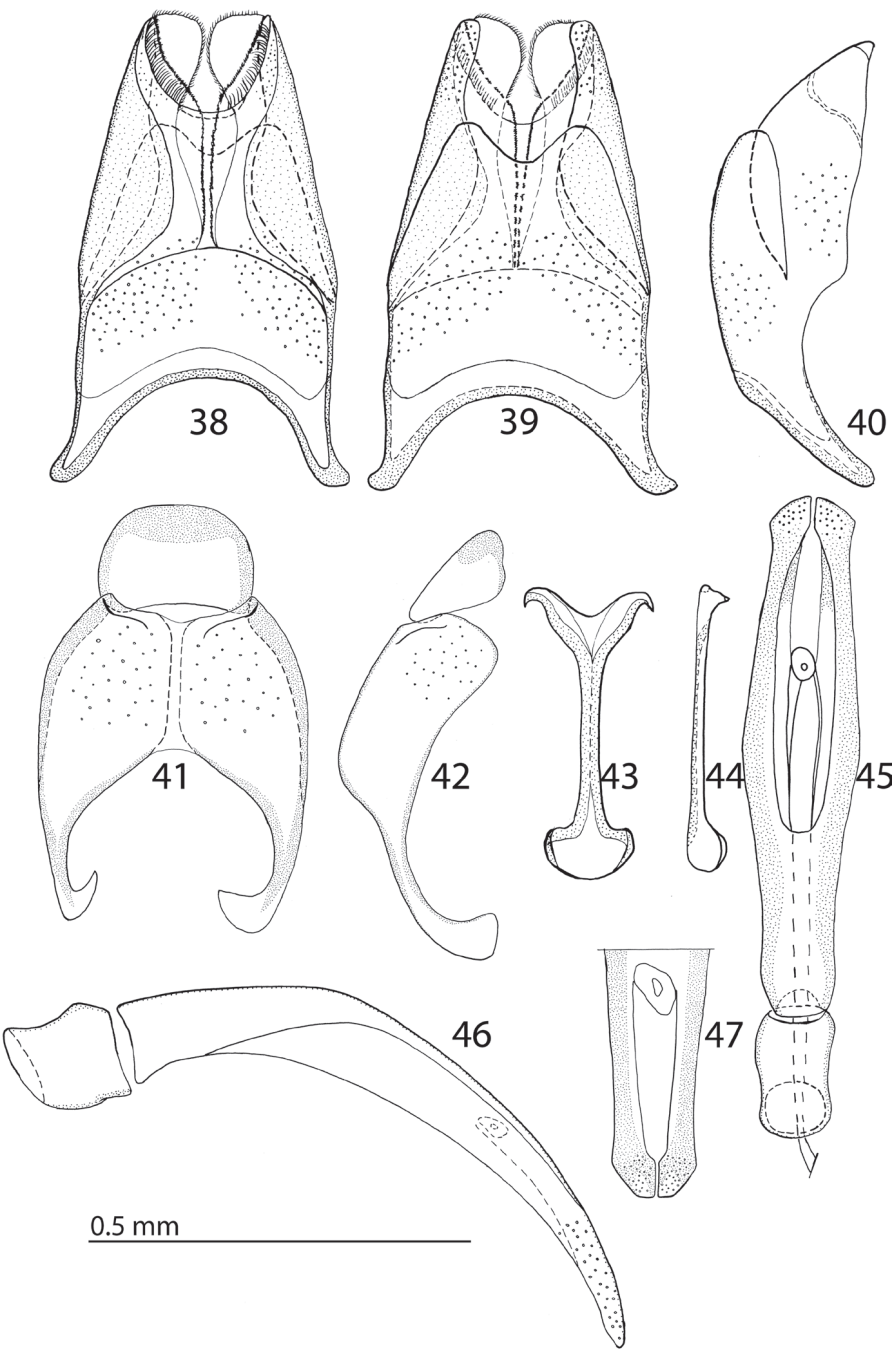
**38** *Reichardtiolus aldhaferi* sp. n., 8^th^ sternite and tergite, ventral view **39** ditto, ventral view **40** ditto, lateral view **41** *Reichardtiolus aldhaferi* sp. n., 9^th^ + 10^th^ tergites, dorsal view **42** ditto, lateral view **43** *Reichardtiolus aldhaferi* sp. n., spiculum gastrale, ventral view **44** ditto, lateral view **45** *Reichardtiolus aldhaferi* sp. n., aedeagus, dorsal view **46** ditto, lateral view **47** *Reichardtiolus aldhaferi* sp. n., apex of aedeagus, frontal view.

Body size: PEL: 2.50–3.25 mm; APW: 0.85–1.15 mm; PPW: 1.80–2.25 mm; EW: 2.00–2.50 mm; EL: 1.50–2.00 mm. Body darker than that of *Reichardtiolus duriculus*, otherwise similar to it. Legs and antennae darker than those of *Reichardtiolus duriculus*; mouthparts similar except mentum, which is on its anterior margin more emarginated than that of *Reichardtiolus duriculus* (compare Figs 4 and 35). Clypeus anteriorly elevated (Fig. 34), with slight median depression, rugosely punctate; frons (Fig. 34) coarsely and densely punctate, medially rugulose-lacunose, with shallow depressions; frontal and supraorbital striae and eyes as in *Reichardtiolus duriculus*. Pronotum slightly less acutely narrowing apically than that of *Reichardtiolus duriculus*; punctuation on pronotal disk sparser than that of *Reichardtiolus duriculus*. Elytra similar to those of *Reichardtiolus duriculus*, but dorsal elytral striae weaker, occasionally striae 3-4 shortened apically, only half as long as striae 1-2 or even evanescent; between 4^th^ dorsal elytral and sutural striae in several specimens punctures scratch-like and surface with variously deep longitudinal wrinkles; rarely with shallow depression between the bases of 4^th^ and sutural elytral striae. Punctuation of elytral disk sparser than that of *Reichardtiolus duriculus*, punctures separated by several times their diameter; in fourth elytral interval occasionally scratch-like. Propygidium and pygidium similar to those of *Reichardtiolus duriculus*, but punctuation denser and coarser in *Reichardtiolus aldhaferi*, although not as dense as in *Reichardtiolus sphingis* (compare Figs 12, 13 and 37). Structure of prosternal process similar to that of *Reichardtiolus duriculus*, but prosternal keel laterally more compressed and setose (compare Figs 10 and 36); carinal prosternal striae occasionally very approximate, medially almost united and difficult to discern; prosternal foveae smaller than those of *Reichardtiolus duriculus*. Mesoventrite sub-square, trapezoidal, punctuation sparse, punctures separated by several times their own diameter; marginal mesoventral stria always complete anteriorly, almost straight; meso-metaventral sutural stria absent, suture distinct. Metaventrite, metepisternum and abdominal ventrites similar to those of *Reichardtiolus duriculus*. Legs as in *Reichardtiolus duriculus*; except denticles of mesotibia that are sparser, thinner and shorter. Male genitalia: 8^th^ sternite (Figs 38–39) strongly sclerotized laterally, apically with pseudopores and a row of short setae and small velum covered with minute setae; 8^th^ tergite (Fig. 39) deeply emarginated apically, on basal half with prominent pores; 8^th^ sternite and tergite fused laterally (Fig. 40). 9^th^ tergite (Fig. 41) well sclerotized along margins, laterally without projection (Fig. 42), apically with two bisinuate strongly sclerotized lines visible from dorsal view, apical half covered with pseudopores, sclerotization of tergite medially divided, two parts held together by weakly sclerotized part; 10^th^ tergite basally faintly inwardly arcuate (Fig. 41). Tips of apical end of spiculum gastrale (Fig. 43) without strongly sclerotized parts, apical end strongly inwardly arcuate, basal end outwardly arcuate. Aedeagus (Figs 45–46) similar to that of *Reichardtiolus duriculus*, but laterally more curved and medially thickened (compare Figs 23 and 46).

#### Differential diagnosis.

As with preceding species.

#### Biology.

Unknown, presumably similar to the congeners, the examined specimens were collected at light in winter months.

#### Distribution.

Saudi Arabia, environs of Riyadh (Fig. 72).

#### Etymology.

Patronymic, named after the head of the entomology department at KSMA, H. M. Al Dhafer.

### Reichardtiolus perses sp. n.

http://zoobank.org/9B800BDD-A4B9-4D0B-BCE1-85CE9859E039

http://species-id.net/wiki/Reichardtiolus_perses

Figs 48 –61

#### Type locality.

Iran, Kerman, Talab.

#### Type material examined.

Holotype, male, side-mounted on triangular point with male genitalia extracted and glued to the same triangular point as the specimen, left protarsus and left mid-leg missing, piece of left elytron from the elytral flank along the elytral base towards the fourth elytral stria chipped out; with the following labels: “♂” (printed); followed by: “Kerman: str. Talab / 19–20.i.[19]01 / N. Zarudny” (printed-written label in Russian); followed by: “Coll. Semenov-Tian-Shansky” (printed); followed by: “ZOOLOGICAL / INSTITUTE RAS / ST. PETERSBURG” (yellow label, printed); followed by: “*Reichardtiolus perses* / sp.nov. HOLOTYPE / Det. T. Lackner 2013” (red label, printed) (ZIN). Paratypes: 1 ♀, ibid (sputter coated with gold) (ZIN); 1 ♀, ibid, but 20.i.[19]01, with an additional written-printed label: “*Exaesiopus* / *duriculus* Rtt. / Reichardt det.” (TLAN).

#### Diagnostic description.

**Figures 48–51.**
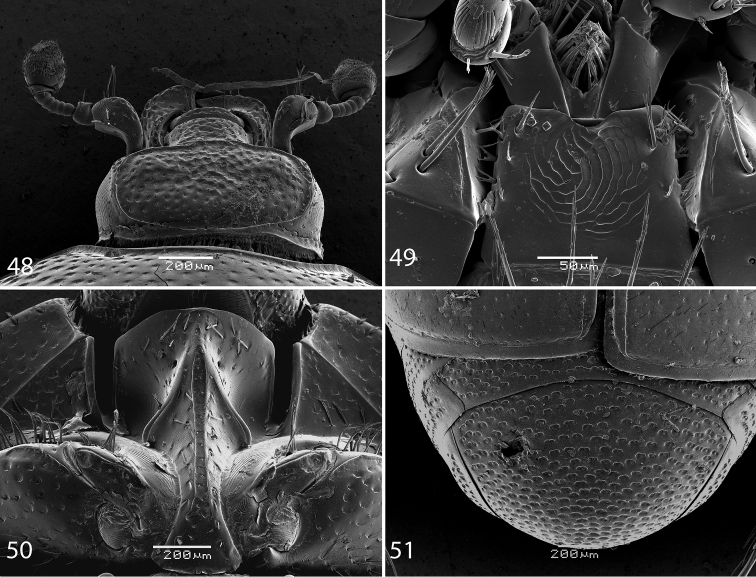
**48** *Reichardtiolus perses* sp. n., head, dorsal view **49** *Reichardtiolus perses* sp. n., mentum, ventral view **50** *Reichardtiolus perses* sp. n., prosternum **51** *Reichardtiolus perses* sp. n., pygidium.

**Figures 52–61.**
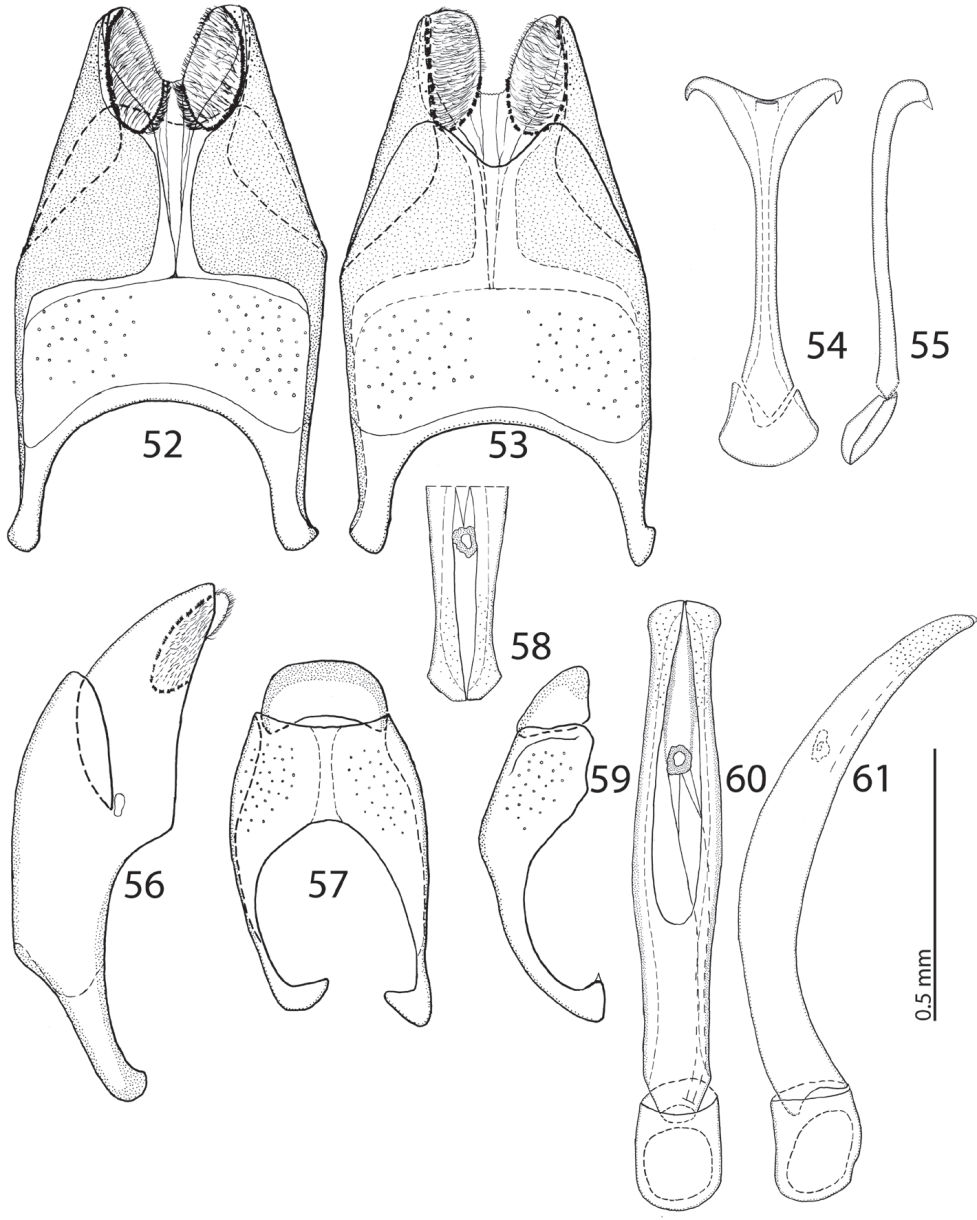
**52** *Reichardtiolus perses* sp. n., 8^th^ sternite and tergite, ventral view **53** ditto, dorsal view **54** *Reichardtiolus perses* sp. n., spiculum gastrale, ventral view **55** ditto, lateral view **56** *Reichardtiolus perses* sp. n., 8^th^ sternite and tergite, lateral view **57** *Reichardtiolus perses* sp. n., 9^th^ + 10^th^ tergites, dorsal view **58** *Reichardtiolus perses* sp. n., apex of aedeagus, frontal view **59** *Reichardtiolus perses* sp. n., 9^th^ + 10^th^ tergites, lateral view **60** *Reichardtiolus perses* sp. n., aedeagus, dorsal view **61** ditto, lateral view.

Body size: PEL: 2.50–3.75 mm; APW: 0.75–1.15 mm; PPW: 1.90–2.75 mm; EW: 2.00–3.00 mm; EL: 1.75–2.50 mm. Body in general (except for *Reichardtiolus pavlovskii*) larger than the rest of congeners, cuticle similar to that of *Reichardtiolus duriculus*; legs, antennae and mouthparts chestnut brown. Mouthparts similar to those of *Reichardtiolus duriculus*, mentum on anterior margin deeply emarginated medially (Fig. 49). Clypeus and frons (Fig. 48) coarsely and densely punctate; frontal stria weakened medially; frontal disk with low protuberances and shallow depressions, very coarsely and densely punctate, especially medially; clypeus margined laterally. Pronotum as in *Reichardtiolus duriculus*, punctuation medially sparser, punctures weak and separated by several times their diameter. Elytra generally similar to those of *Reichardtiolus duriculus*; punctuation of pygydium generally denser than that of *Reichardtiolus duriculus* (compare Figs 12 and 51). Prosternal process flattened to slightly concave, compressed laterally; carinal prosternal striae approximate, complete; prosternal foveae small. Mesoventrite sub-quadrate, marginal stria anteriorly complete; punctuation sparser than that of *Reichardtiolus duriculus*, punctures separated by several times their diameter; meso-metaventral stria absent, in case of one specimen substituted by a string of punctures. Metaventrite, metepisternum and abdominal ventrites similar to those of *Reichardtiolus duriculus*. Legs similar to those of *Reichardtiolus duriculus*, *Reichardtiolus sphingis*, and *Reichardtiolus aldhaferi*. Male genitalia: 8^th^ sternite (Figs 52–53) strongly sclerotized, apically with dense row of short setae and setose velum; 8^th^ tergite apically with deep emargination, on basal half with numerous pores and pseudopores (Fig. 53). Sclerotization of 9^th^ tergite divided medially (as in *Reichardtiolus aldhaferi*), on apical half with pores and pseudopores; 10^th^ tergite inwardly arcuate on its basal margin. 9^th^ tergite on apical third with faint, weakly sclerotized bisinuate line, visible only from lateral view (Fig. 59). Spiculum gastrale (Fig. 54) on apical end inwardly arcuate (although not as deeply as with *Reichardtiolus sphingis* or *Reichardtiolus aldhaferi*), with a unique sclerotized ring medially; basal end of spiculum gastrale outwardly arcuate. Aedeagus generally most similar to that of *Reichardtiolus sphingis*, but blunt apically (compare Figs 31 and 58).

#### Differential diagnosis.

*Reichardtiolus perses* is the second largest species of the genus (after *Reichardtiolus pavlovskii*) and externally very similar to *Reichardtiolus duriculus*, *Reichardtiolus aldhaferi*, and *Reichardtiolus sphingis*, differing from them mainly by the structure of male terminalia. From the largest species of the genus, *Reichardtiolus pavlovskii* it differs by the same characteristics as the preceding three species.

#### Biology.

Unknown, presumably similar to its congeners.

#### Distribution.

Iran, environs of Kerman (Fig. 72).

#### Etymology.

The name of this new species means “Persian”. It is a noun in apposition in the nominative singular form.

### Reichardtiolus pavlovskii

Kryzhanovskij, 1959

http://species-id.net/wiki/Reichardtiolus_pavlovskii

Figs 62–71

Exaesiopus pavlovskii Kryzhanovskij, 1959: 216, fig 1.
Reichardtiolus pavlovskii : Kryzhanovskij in Kryzhanovskij and Reichardt (1976): 239, 240; Mazur (1984): 103; Mazur (1997): 265; Mazur (2004): 96; Mazur (2011): 210.

#### Type locality.

Turkmenistan, Badkhyz Nature Reserve.

#### Type material examined.

Holotype, female, side mounted on a triangular mounting point: “Yu. V. [=Yugo-Vostochnyj, South-Eastern] Turkm. [=Turkmenistan], Badkhyz / 12 km W Kala-i-Mor / 31.iii.1957 G. Medvedev” [written]; “Barkhannye peski [= moving sands]” (written); “*Exaesiopus* / (*Reichardtiolus*) / *pavlovskii* m., typ. / O. Kryzhano- / vskij det [1]958” (printed-written); “Holotypus / *Exaesiopus* / *pavlovskii* Kryzh.” (red label, written); “Zoological / Institute RAS / St. Petersburg” (yellow printed label); “09-068” (yellow pencil-written label), added by the author (ZIN).

#### Re-description.

**Figures 62–71.**
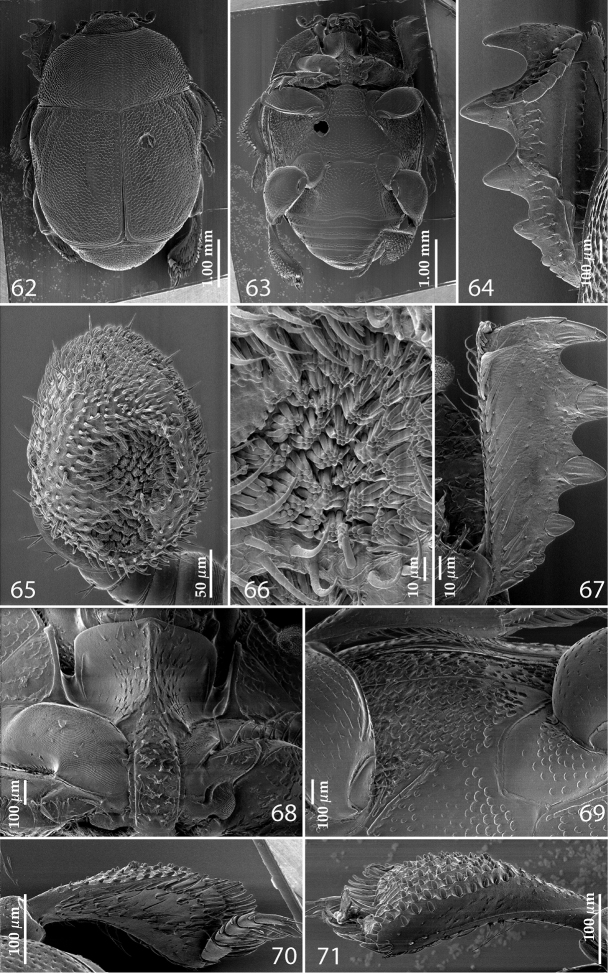
**62** *Reichardtiolus pavlovskii* (Kryzhanovskij, 1959) habitus, dorsal view **63** *Reichardtiolus pavlovskii* (Kryzhanovskij, 1959) habitus, ventral view **64** *Reichardtiolus pavlovskii* (Kryzhanovskij, 1959) protibia, dorsal view **65** *Reichardtiolus pavlovskii* (Kryzhanovskij, 1959) antennal club, ventro-lateral view **66** *Reichardtiolus pavlovskii* (Kryzhanovskij, 1959) detail of the sensory area of the antenna **67** *Reichardtiolus pavlovskii* (Kryzhanovskij, 1959) protibia, ventral view **68** *Reichardtiolus pavlovskii* (Kryzhanovskij, 1959) prosternum **69** *Reichardtiolus pavlovskii* (Kryzhanovskij, 1959) lateral disk of metaventrite + fused metepisternum **70** *Reichardtiolus pavlovskii* (Kryzhanovskij, 1959) metatibia, dorsal view **71** *Reichardtiolus pavlovskii* (Kryzhanovskij, 1959) ditto, ventral view.

Body size PEL: 4.25 mm; APW: 1.25 mm; PPW: 3.20 mm; EL: 3.50 mm; EW: 3.00 mm. Body (Figs 62–63) rectangular oval, strongly convex, pronotum somewhat narrower than elytra, cuticle dark brown to black, elytra somewhat lighter, without metallic luster, entire dorsal surface rugulose-lacunose; legs, mouthparts and antennae light to dark brown, antennal club black.

Antennal scape not particularly thickened, punctate dorsally, punctures with numerous long setae; club (Fig. 65) oval, slightly depressed dorso-ventrally; without visible articulation, entire surface with thick short yellow sensilla intermingled with sparse longer erect sensilla, ventrally with two large round sensory areas (Figs 65, 66); sensory structures of antennal club not examined. Mouthparts: mandibles stout, densely punctate, dorso-lateral area with sparse short setae, acutely pointed; labrum convex with two labral setae growing out from each labral pit; square-shaped, anterior angles produced, anterior margin with deep median excavation, surface around it with four longer setae; lateral margins with double row of shorter ramose setae; disc of mentum imbricate; other parts of the mouth not examined.

Clypeus sub-quadrate, coarsely punctate, slightly depressed medially and slightly carinate laterally; frontal stria carinate, interrupted anteriorly, continuous with weakly carinate supraorbital stria; frontal disc rugulose-lacunose; eyes flattened, but visible from above.

Pronotal sides (Fig. 62) on basal two-thirds moderately convergent anteriorly, strongly convergent anteriorly on apical third, apical angles blunt; pronotal foveae absent; marginal pronotal stria complete, carinate, slightly weakened behind head; disc of pronotum completely with deep coarse elongate punctures separated by less than half their diameter forming rugulose-lacunose wrinkles medially; pronotal hypomeron with short yellow setae; scutellum very small, visible.

Elytral humeri slightly prominent; elytra widest at humeri; elytral epipleura in large punctures; marginal epipleural stria complete, surface between it and elytral margin smooth; marginal elytral stria straight and carinate, continued as somewhat weakened complete apical elytral stria continuous with sutural elytral stria. Humeral elytral stria faintly impressed on basal third; inner subhumeral stria present as a median fragment; dorsal elytral striae vaguely impressed, almost obliterated under coarse rugulose-lacunose punctuation, only first and second dorsal striae distinguishable, not reaching elytral midpoint apically, third and fourth striae faint, shorter than first and second; sutural elytral stria faintly impressed, abbreviated at basal tenth, complete to apex, continuous with apical elytral stria; entire elytral disc (with exception of elytral humeri) rugulose-lacunose.

Propygidium largely covered by elytra; its punctuation similar to that of elytral disc; pygidium also densely and coarsely punctate; punctures with minuscule setae.

Anterior margin of median portion of prosternum (Fig. 68) projected medially, setose; prosternal foveae absent; marginal prosternal stria present laterally and as extremely short apical rudiment; prosternal apophysis constricted between procoxae, rugulose-lacunose, setose, prosternal process thence strongly compressed, knife-like, setose, surface imbricate, dorso-medially with numerous setiferous punctures; vestiges of carinal prosternal striae present on prosternal apophysis; lateral prosternal striae present as faint rudiments, almost invisible.

Anterior margin of mesoventrite with slight median projection; discal marginal mesoventral stria complete; disc of mesoventrite convex, rugulose-lacunose; meso-metaventral suture straight, thin; meso-metaventral sutural stria undulate; intercoxal disc of metaventrite (Fig. 69) depressed medially; with sparser and finer punctuation than that of mesoventrite, punctures separated by two-three times their diameter; lateral metaventral stria straight, shortened; lateral disc of metaventrite slightly excavate, with dense deep setiferous punctures; metepisternum (Fig. 69) with similar setiferous punctures; fused metepimeron with sparser punctuation; lateral metepisternal stria complete, deeply impressed.

Intercoxal disc of first abdominal ventrite completely striate laterally; completely covered with punctuation; punctures similar to those of disc of metaventrite.

Protibia (Figs 64, 67) dilated, outer margin with three large widely-spaced distal teeth topped by large triangular denticle, diminishing in size in proximal direction, followed by two smaller proximal denticles; setae of outer row thin, sparse and short; setae of median row similar to those of outer row; protarsal groove shallow; anterior protibial stria carinate, almost complete; protibial spur small, straight, growing out near tarsal insertion; outer part of posterior surface of protibia (Fig. 67) almost smooth, only with scattered microscopic denticles, demarcation line between outer and median of posterior surface non-existent; posterior protibial stria absent, near inner protibial margin a dense row of strongly sclerotized long setae present; inner margin with sparser row of thinner setae.

Mesotibia slightly thickened, outer margin with row of approximately ten long denticles increased in size apically; setae of outer row dense and long, strongly sclerotized, longer than denticles on outer margin; setae of median row absent; posterior mesotibial stria absent; anterior surface of mesotibia with additional two-three dense rows of short denticles; anterior mesotibial stria complete, terminating in several minute denticles; mesotibial spur short; apical margin of mesotibia with a row of about five short denticles; first and second tarsomere ventrally with four long, strongly sclerotized setae; third and fourth tarsomeres with only two such setae; fifth tarsomere devoid of setae ventrally; claws of apical tarsomere slightly bent, longer than tarsomere itself; metatibia (Fig. 70) much more thickened and dilated than mesotibia, outer margin and posterior surface similar to that of mesotibia; anterior surface of metatibia completely covered with six-seven rows of short, stout denticles (Fig. 71).

Male unknown.

#### Differential diagnosis.

Externally somewhat similar to its congeners, it is, however, the most readily distinguishable species of the five. Body (Figs 62–63) larger than in all other congeners (up to 4.25 mm in *Reichardtiolus pavlovskii*, whereas other *Reichardtiolus* species attain maximal body length of 3.75 mm), cuticle dark brown to black, entire dorsal surface rugulose-lacunose, whereas the dorsal surface of the other species is mostly chestnut brown and punctate, never rugulose-lacunose. Dorsal elytral striae (Fig. 62) of *Reichardtiolus pavlovskii* are vaguely impressed, almost obliterated under coarse rugulose-lacunose punctuation, only first and second dorsal striae distinguishable, while with the rest of congeners they are usually distinct. This species differs likewise from the rest of its congeners by the structure of the prosternal keel (compare Figs 10–11, 36, 50 and 68), which is projected medially, strongly compressed, almost knife-like, lacking foveae, and with only vestigial striae. *Reichardtiolus pavlovskii* also differs from the other species by the lateral disc of the metaventrite and fused metepisternum (Fig. 69) that are covered with almost confluent setiferous punctures, whereas the punctures are not confluent in *Reichardtiolus duriculus*, *Reichardtiolus perses*, *Reichardtiolus aldhaferi* or *Reichardtiolus sphingis*. The protibia (Figs 64 and 67) is similar to the other three species, but adorned with three short teeth topped by acute large triangular denticle (instead of two) followed by one shorter denticle entombed in protibial margin and one more microscopic denticle. The mesotibia on its anterior surface has an additional two-three dense rows of short denticles instead of the single row present in *Reichardtiolus duriculus*, *Reichardtiolus sphingis*, *Reichardtiolus perses* and *Reichardtiolus aldhaferi*; the metatibia (Figs 70–71) is much more thickened and dilated than those of the other four species; the anterior surface of metatibia has six-seven rows of short stout denticles as opposed to only two rows in *Reichardtiolus duriculus*, *Reichardtiolus sphingis*, *Reichardtiolus perses* and *Reichardtiolus aldhaferi*. Unfortunately, the only examined specimen is a female so the male genitalia could not be compared to those of other species.

#### Biology.

Found in the sand under *Tamarix* (Kryzhanovskij & Reichardt, 1976).

#### Distribution.

**Figures 72.**
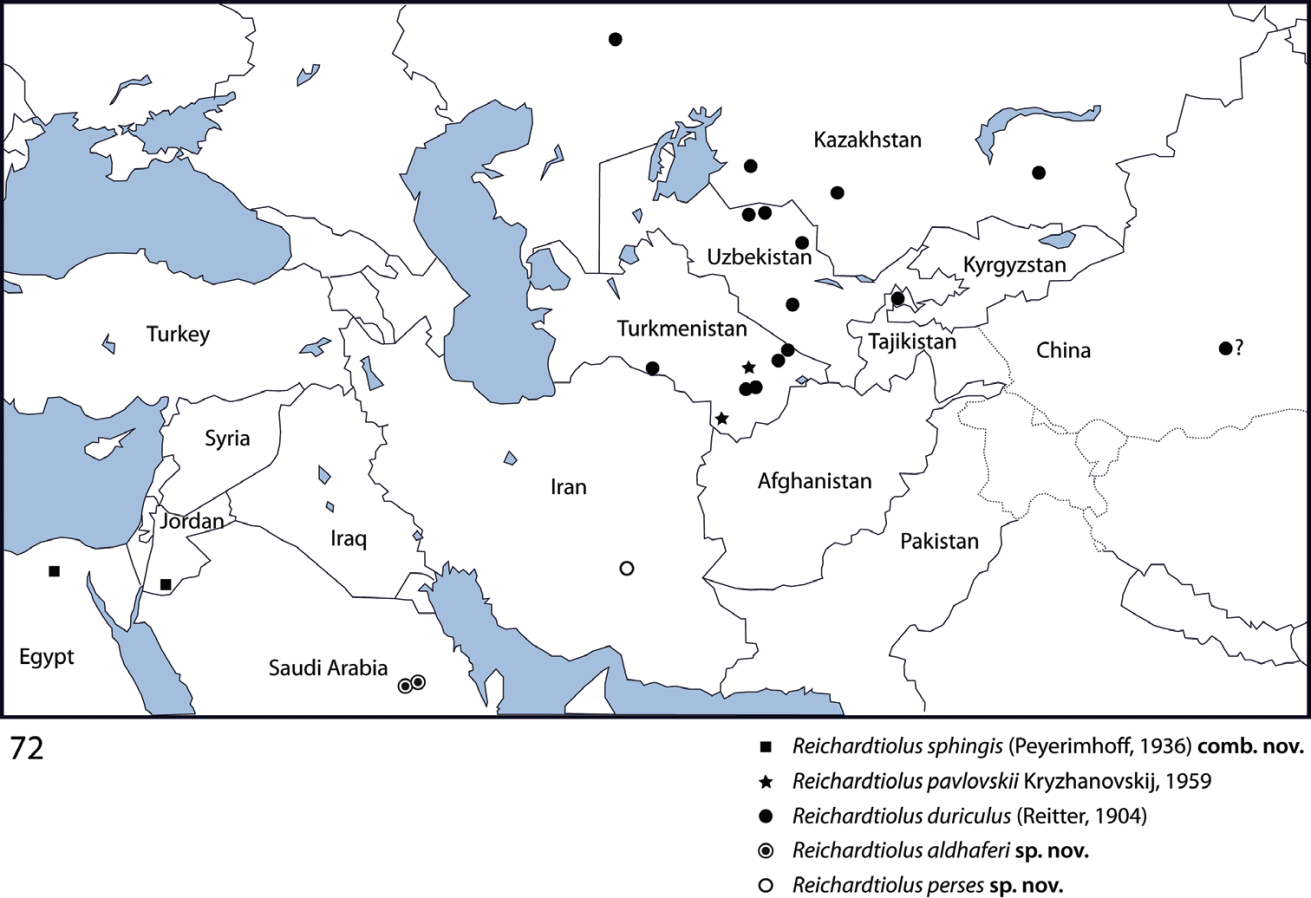
Distributional map of *Reichardtiolus* Kryzhanovskij, 1959.

So far known only from two places in Turkmenistan: about 40 km north of Mary, eastern Turkmenistan and Badkhyz Nature Reserve, southeastern Turkmenistan (Fig. 72).

#### Remarks.

Kryzhanovskij (1959), in his original description, omitted the character of the prosternal striae, and in the Fauna USSR (Kryzhanovskij and Reichardt 1976) he provided a brief re-description of this species but omitted the prosternum altogether, pointing only to the greater size and surface of the dorsal side of body as distinguishing characters for separating *Reichardtiolus duriculus* from *Reichardtiolus pavlovskii*. *Reichardtiolus pavlovskii* is, according to Kryzhanovskij in Kryzhanovskij and Reichardt (1976) known only from two females and I have only examined one of them, the holotype. The repository of the second specimen of this rare species is unknown. Although *Reichardtiolus pavlovskii* is morphologically rather different from the other species of the genus, I am hesitant to erect a new genus for it, especially since no male is available and the male terminalia could not be examined.

### Key to the species of the genus *Reichardtiolus*

**Table:** 

1(2)	Metatibia on anterior surface (Fig. 71) with more than 5 dense rows of tiny denticles; protibia on outer margin with three short teeth topped by denticle (Fig. 64), followed by one more small tooth embedded in the outer margin topped by a denticle and a minuscule denticle; a large species (4.25 mm) (Turkmenistan)	*Reichardtiolus pavlovskii* (Kryzhanovskij, 1959)
2(1)	Metatibia on anterior surface with one or two sparse rows of tiny denticles (for fig. see Lackner 2010: fig 602); protibia on outer margin with two short teeth topped by denticle (for fig. see Lackner 2010: fig 603), followed by one more small tooth embedded in the prosternal margin topped by a denticle and a minuscule denticle; smaller species (up to 3.80 mm).
3(4)	Mentum almost without emargination on anterior margin (Fig. 4), 8^th^ tergite apically almost straight (Fig. 15); spiculum gastrale apically only faintly inwardly arcuate (Fig. 19), species from middle Asia	*Reichardtiolus duriculus* (Reitter, 1904)
4(3)	Mentum anteriorly with moderately deep to deep emargination (Fig. 5), 8^th^ tergite apically deeply emarginate (see for example Fig. 39); spiculum gastrale apically strongly inwardly arcuate (see for example Fig. 43); species from Near East, Iran.
5(6)	Aedeagus strongly curved from lateral view (Fig. 46), thickened medially (Fig. 45); species from Saudi Arabia	*Reichardtiolus aldhaferi* sp. n.
6(5)	Aedeagus only moderately curved from lateral view (Figs 33, 61), not particularly thickened medially (Figs 32, 60); species from Egypt, Jordan and SW Iran
7(8)	Basal margin of 10^th^ tergite moderately inwardly arcuate, without a prominent incision (Fig. 57), both tips of apical end of spiculum gastrale without strongly sclerotized parts (Fig. 54), sclerotization of 9^th^ tergite medially divided (Fig. 57), species from SW Iran	*Reichardtiolus perses* sp. n.
8(7)	10^th^ tergite on basal margin with median incision (Fig. 26), both tips of apical end of spiculum gastrale with strongly sclerotized parts (Fig. 29), sclerotization of 9^th^ tergite undivided medially (Fig. 26), species from N Egypt and S Jordan	*Reichardtiolus sphingis* (Peyerimhoff, 1932)

## Discussion

*Reichardtiolus* is a small psammophilous Saprininae genus currently comprising five species: *Reichardtiolus duriculus*, *Reichardtiolus sphingis*, *Reichardtiolus aldhaferi*, *Reichardtiolus perses* and *Reichardtiolus pavlovskii*. Although the four former species are morphologically very similar and undoubtedly related, the latter species *Reichardtiolus pavlovskii* is rather different from the rest and characterized by several autapomorphies, e.g. rudimentary sets of prosternal striae, absence of prosternal foveae, and more than five rows of densely set short denticles on the anterior surface of metatibia. Its protibia is also different from those of *Reichardtiolus duriculus*, *Reichardtiolus sphingis*, *Reichardtiolus perses* or *Reichardtiolus aldhaferi* by having an extra tooth on its outer margin. The four morphologically similar species apparently represent allopatric congeners all sharing a rather recent common ancestor, since they only differ in minute details most evident in their male genitalia. It is possible that their common ancestor came from the deserts of middle Asia, and subsequently speciated in the arid regions of North Africa, Near East, and Iran in search for new habitats as a form of adaptive radiation. All five species seem to be well adapted to the psammophilous way of life with thickened femora and tibiae, enlarged protibiae with large triangular teeth each topped by a denticle, as well as having the underside of the body covered with vestiture.

Phylogenetically speaking, the type species of the genus has been recovered in the recently performed cladistic analysis of the author (Lackner, unpublished) as a member of a large unresolved clade of taxa that all share a single unique synapomorphy of a single, stipe-shaped vesicle inside the internal-distal part of the antennal club, as well as several other, weaker synapomorphies. However, the species *Reichardtiolus pavlovskii*, which was also included in the analysis, has been recovered rather distant from the type species of the genus, *Reichardtiolus duriculus*. Because of the low resolution of the morphology-based cladogram, and absence of a male specimen of *Reichardtiolus pavlovskii* I decided not to alter the generic rank of the latter species. The members of the genus *Reichardtiolus* cover a rather vast area (Fig. 72) from the Chinese Xinjiang province in the east to the Egyptian locality in the west, from the Kazakh localities in the north to the Saudi Arabian localities in the south. Such a vast area likely houses further undescribed species of *Reichardtiolus* and it is hoped that this study shall encourage their discovery by fellow entomologists.

## Supplementary Material

XML Treatment for Reichardtiolus

XML Treatment for Reichardtiolus duriculus

XML Treatment for Reichardtiolus sphingis

XML Treatment for Reichardtiolus aldhaferi

XML Treatment for Reichardtiolus perses

XML Treatment for Reichardtiolus pavlovskii
